# Spiritual care in the dementia ward during a pandemic

**DOI:** 10.1007/s11017-024-09666-2

**Published:** 2024-05-16

**Authors:** Talitha Cooreman-Guittin

**Affiliations:** Université de Fribourg, Fribourg, Switzerland

**Keywords:** Spiritual care, Dementia, Pandemic, Residential care

## Abstract

The Covid-19 pandemic and the repeated lockdowns have caused substantial spiritual and existential suffering, not the least for persons with dementia who may have had more difficulties than others in grasping the reality of what was going on. Therefore, it is important to address spirituality within this sector of the population when considering global health and ethics and technology in a pandemic outbreak. This contribution starts firstly with a definition of spirituality and spiritual care. Secondly, based on the works of Elizabeth MacKinlay and Laura Dewitte, the article demonstrates how spirituality can be nurtured in the dementia ward through “spiritual reminiscence.” Finally, I briefly reflect on how spiritual care in the dementia ward was affected by the Covid-19 pandemic.

## Introduction^1^

It may come as a surprise to some of the readers of this journal to find a contribution about spiritual care for people with dementia in a volume concerning issues of global health ethics during a pandemic outbreak. However, the pandemic and repeated consecutive lockdowns caused substantial spiritual and existential suffering, not least for persons with dementia who may have had more difficulties than others grasping the reality of what was going on [2]. Therefore, it is important to address the subject of spirituality with this sector of the population when taking into account global health and ethics and technology within the framework of a pandemic outbreak.

To introduce this topic, let me share a short experience of my work in the dementia ward.

The old woman sits at a table in the middle of the dementia ward and sings "Kyrie Eleison" for hours on end with her eyes closed. Her singing exasperates the staff, but there seems to be nothing that can stop her. I ask the ward nurse if I can have a chat with her, but I am told she has an MMSE^2^ of 11, so she’s in an advanced stage of dementia, incapable of much conversation anymore. I ask the nurse if I can try. She shrugs. “Do you as you please,” she says. The old woman is still singing. I ask her if I can sit down. She nods and goes back to her "Kyrie". Then I ask if she wants to pray with me. "Oh yes... " I begin a Hail Mary which she recites with me, her eyes closed. "Do you want to pray the Lord’s Prayer?" She nods and opens her hands. Together we pray Our Father, after which she continues: "Credo in unum Deum" ... she looks at me expectantly. I was born after Vatican II - Latin does not come easily to me without the reassuring presence of a community. Nevertheless, I sing with her: "Patrem omnipotentem, factorem caeli et terrae" ... and already I hesitate. "It's more complicated in Latin, isn’t it?" she says mischievously, and she chuckles - and there she goes again with her Kyrie incantations.

In my experience, most medical doctors attribute what happened between the old woman and me to ‘procedural memory’ alone, with no deeper meaning. However, I find this very difficult to agree with—and several of my colleague/researchers also believe that what happened here is not without importance. Research at the KU Leuven in Belgium has shown that, even in advanced stages of dementia, people still give meaning to their lives [3]. I shared a beautiful, deeply religious and spiritual moment of prayer with the old woman, and I have the audacity to believe that, for her, the joy was shared and that she was fully aware of what we were doing. Scottish theologian John Swinton has argued that procedural memory functions as a real pathway that puts people with dementia in the presence of Jesus Christ [4]. All those years of prayer, all those hours spent at Mass, all those readings of the Gospel bear fruit when these people celebrate *in their body* the memory of God. This may happen through procedural memory, but that does not imply that it has no significance. It does not matter that the old woman does not remember our prayer five minutes later. In God's time, there is only the present.

In this contribution, I will first define how I understand spirituality and spiritual care. Secondly, I will show how spirituality can be nurtured in the dementia ward through “spiritual reminiscence” and finally I will look at how spiritual care was affected by the Covid-19 pandemic.

## Definition

As a theologian, I consider that to be human involves biological, psychological, social, and spiritual dimensions. Needless to say, the most controversial of these four dimensions is the last one. Here, I will not go into the controversies about what spirituality is and what it is not. Rather, in the framework of this contribution, I suggest working with what is commonly referred to as Christina Puchalski’s ‘consensus’ definition:

Spirituality is a dynamic and intrinsic aspect of humanity through which persons seek ultimate meaning, purpose, and transcendence, and experience relationship to self, family, others, community, society, nature, and the significant or sacred. Spirituality is expressed through beliefs, values, traditions, and practices [5, p. 646].

Though this definition is interesting to work with, I find it too focused on cognition, because it intrinsically links spirituality to meaning-seeking. Persons with cognitive challenges, in advanced stages of dementia for example, or persons with profound intellectual disabilities, might not always be able to reflect cognitively on the meaning of life. This does not imply, however, that they are void of spirituality or that their life has no meaning. I suggest combining Puchalski’s consensus definition with the definition of Belgian ethicist Dominique Jacquemin, who separates the notion of spirituality from the search for meaning:

Spirituality is the movement of existence of the human subject. This movement of existence [...] is made up of three or four dimensions that are intrinsically linked and in constant interaction: the bodily, the psychic dimension, the ethical dimension as the aim for a good life and for some the religious / transcendent dimension [6, p. 126].

For Jacquemin, each of these dimensions constitutes a possible way to encounter a person’s spirituality. Interacting with the body, taking care of a person’s body, can have a deeply spiritual meaning, even when the persons engaged are not explicitly looking for an ultimate meaning. Having said this, I do not want to dismiss the search for meaning with people who experience dementia on the pretext that they can no longer think abstractly. Recent studies show quite the opposite. In her doctoral research *Losing memory, losing meaning*, Laura Dewitte clearly demonstrates that there is no correlation between the perceived sense of meaning and the cognitive abilities of elderly people with dementia. In her research, Dewitte states that persons with a low MMSE score can still indicate that they feel their life has meaning. For older adults in residential care, with or without dementia, many sources of well-being remain highly meaningful and are directly related to the experience of meaning in life. For elderly persons with dementia, continued investment in moments that foster personal growth and family relationships seem to be especially valuable [3].

The research also shows that both relatives and healthcare professionals tend to overestimate the importance of cognitive abilities in how people evaluate their lives. The people around the person with dementia will often have a negative assessment of the quality of life of this person, even if the person with dementia clearly express that they are happy. The following example illustrates this phenomenon:

I ask Mr. F. if he is happy - and he answers, very convinced: "YES." I ask him: "What makes you happy?" He says: "My family, my children." His daughter, who is sitting next to me, leans over and whispers in my ear: "Don't listen, he doesn't know what he is saying anymore."^3^

Dewitte emphasises that continuing to experience meaning in one's life is still possible, even for persons with dementia. She says that it is absolutely necessary in residential care to promote personal fulfilment. Finally, she pleads for an awareness of our collective responsibility in supporting people with dementia.

So how can one explain how meaning might be preserved when cognitive skills decline? One possible explanation is that people with dementia experience a more *felt sense of meaning*, a physically and corporeally-felt meaning; more implicit and holistic. It is a sense of meaning resulting from a dynamic synthesis of emotional and cognitive processes, but which does not require that all cognitive functions are fully intact. This *bodily felt sense of meaning* could coincide with what Jacquemin calls ‘spirituality.’ At this stage I want to emphasize that spirituality is not necessarily religious, and that it is not the prerogative of priests, chaplains, or faith communities to care for someone’s spiritual wellbeing, even though more often than not these are the persons who are in charge of effectively organizing spiritual care.

Indeed, there is a growing interest in healthcare settings in Belgium, France, and Switzerland for non-religious *spiritual care,* which is considered integral to compassionate, person-centred healthcare and should be a part of routine care. In a perfect world, all healthcare providers would be knowledgeable about the options for addressing patients' spiritual distress and they would enlist spiritual resources to respond to spiritual needs. In my own field of study, theological ethics related to persons with cognitive challenges, there is quite a lot of research going on into the spiritual lives of these persons and how to provide spiritual care for them [8–10].

In the English-speaking world there is a whole host of spiritual care resources for people with dementia. This literature is meant primarily for chaplains of Christian faith communities, with the aim of providing pastoral care for people with dementia. These manuals address the spiritual (understood in this context as religion-related) needs of people with cognitive impairments; they seek to provide chaplains with tools for communicating God's love to people at all stages of the disease. Since this literature is identified as 'Christian' and is mostly theological in nature, it is not always accessible to healthcare providers. One can say that there is a gap in literature on non-religious spiritual care for persons with dementia—though the French-speaking network RESSPIR^4^ [11] is working on these issues.

The Australian researcher Elizabeth MacKinlay has been experimenting for almost 20 years with a method that combines the intuitions of reminiscence therapies for individuals with dementia with the insights of spiritual care. Her method is called *Spiritual Reminiscence* [12] and is not based on religious beliefs. In the following paragraphs I briefly explain her method.

## Spiritual care in the dementia ward

Spiritual reminiscence is part of the theoretical framework of reminiscence therapies that have been developed in the healthcare sector for the past thirty years. Its aim is to improve the wellbeing of patients and to bring back memories of the past through multiple activities. Reminiscence therapy is used in residential care settings as a non-medicinal therapy by psychotherapists or by social workers, in art therapy, or in occupational therapies. This form of therapy involves activities such as memory boxes, wonderfoons [13], animal-assisted therapy, stimulation of the five senses, etc. All these therapies seek to bring emotional wellbeing, to build coherence in a life story, and to find meaning in life events. They are obviously very close to the objectives of spiritual care in responding to the need for relationship and taking into account the totality of the person's experience of existence.

MacKinlay's spiritual reminiscence method combines the intuitions of reminiscence therapy and spiritual care. The method does not seek to reactivate buried memories, but rather to take stock of the demented person's current feelings about their life. As the present is often the only time accessible to the person with dementia, the method explores the present experience of the person, which sometimes provokes the emergence of distant memories.

Each session is structured around a theme. I was able to experiment with the short version of the method — particularly well suited for people in more advanced stages of dementia – which is divided into six themes that I slightly adapted to the French context: Meaning of life, Relationships, loneliness, Hopes and worries, Growing old, Faith, Friendship, and the Church. MacKinlay’s method invites people with dementia to explore what gives meaning to their life in the present. She writes: “Meaning is at the centre of what it is to be human and loss of meaning can be an important factor in grief and depression” [14, p. 17]. Here, in a nutshell, is how each session goes:

I enter the ward at 14:30 – I greet all the residents and the personnel collectively and I install at the end of the ward a table with a cloth, a plant, a sign with the topic of today’s conversation, my I-pod with some music, and 5 comfortable chairs. Then I go and see the residents individually, I present myself (every week), I tell them why I am here and ask if the person wants to join the conversation. It is usually 15:15 when I have a group of 5 or 6 residents around the table. I again present myself and explain once more why I am here and I introduce today’s topic – most of the time someone has already read aloud what’s on the sign. I play a song vaguely related to the topic and then I have two or three questions: e.g.: What is it like to grow old? (One lady replied: “De ça on ne parle pas^5^”) How does memory loss affect you? Who helps you when you feel down? What do you hope for now?

This may come as a surprise, but for a person with dementia it is often easier to answer the question “What do you hope for now?” than “What did you eat for lunch?”. And yet, the latter is the question that people who are ill are most often asked.

It takes time to answer these questions—participants will initially reply to me rather than to the group. But when confidence grows, when everyone is relaxed and enjoying it, participants will eventually interact with one another. We always end on a positive note. We sing and sometimes we pray (this depends on who is in the conversation), and we say goodbye. It is about 16:00 when we end the session. I then follow up individually with any resident who feels like chatting. I leave the ward at 17:00.

Spiritual reminiscence has no therapeutic goal—it has not cured anyone’s dementia. What it does is to recognize both the persons with dementia and the moderator as valued partners in a conversation. This changes the way people look on each other, on themselves, and the way bystanders look on them. This kind of spiritual care values and stimulates a dimension that is often neglected in residential care facilities for persons with dementia. But what happened in March 2020?

## Spirituality and the pandemic

When the pandemic struck in March 2020 and the world went into lockdown, the sole focus of healthcare systems (at least in France, Belgium, and Switzerland) suddenly became the survival of the body in its purely biological dimension. Patients in hospitals and people in residential care were cut off from their relatives and even from their fellow residents. The social dimension of their existence was completely neglected. The acute work overload of medical personnel—adding to an existing chronic overload—made attention to older persons’ mental health scarce, and the psychological^6^ dimension of older people’s wellbeing was often neglected. The *transcendent* dimension simply disappeared from their healthcare. The Resspir-network has published an extremely well-documented book on this issue [15]. Being considered non-essential, chaplains, volunteers, and people of faith (the main providers of spiritual care) were temporarily ousted out of residential care settings. If spirituality is about connectedness, then people with dementia in residential care were seriously spiritually neglected. It remains to be seen how the repeated lockdowns impacted on the long-term spiritual wellbeing of elderly adults with dementia in residential care. From my discussion with doctors, nursing assistants, and relatives, it seems that a lot of residents reacted with lassitude and non-comprehension, some did not behave any differently, while others grieved. Research carried out in France in 2021 with 72 Alzheimer's patients by a team of French and Australian researchers led by Professor Mohamad El Haj from Nantes University, in conjunction with the geriatric unit of the Tourcoing hospital, showed that the repeated lockdowns caused a significant increase in depression [16].

The pandemic and the lockdowns were also very hard on friends and relatives outside the residential settings: to see their loved ones locked up without being able to touch them was extremely frustrating. Video calls have their limits and are not always appropriate for persons with dementia who often relate through physical contact. This need to touch is called “skin-hunger” and research into covid-related “touch-starvation” is only at an early stage [17]. But, returning to Jacquemin’s definition of spirituality as a movement of existence between the corporeal, the psychological, the social and transcendent dimensions of a person, with the body as a possible gateway to human spirituality, then it follows that depriving people of social contact, psychological assistance, and skin-to-skin-contact, cuts them from all possible spiritual stimulation for months on end, and it is unknown what the consequences of these deprivations are.

Fortunately, all is not pain and sorrow. Several assistants in nursing homes have told me that individual relationships with residents deepened during the pandemic. Being unable to minister to groups, these assistants resorted to one-on-one meetings with their residents, which enabled more personal encounters and a better knowledge of each other. One assistant said this led to a sustainable change in the way she organizes her work.

Consider the testimony of the charity organization *Time to Talk Befriending* [18]—an English intergenerational befriending project with two chaplains and seven volunteers. During the lockdowns, they continued to offer telephone chaplaincy to 42 individuals, several of whom have dementia. *Time to Talk* was delighted to observe that regular 15-min telephone calls offering spiritual care to people with dementia worked very well. A simple question like, ‘What can you see from your window?’ led to wonderful conversations.

## Conclusion

In conclusion, I would like to reaffirm that caring for human being’s spiritual lives is important if they are to flourish; this applies to all human beings, whatever their cognitive abilities. A qualitative systematic review conducted in 2021 [19] concluded that spirituality is still not formally addressed in elderly populations affected by dementia. Professionals still do not feel confident to integrate spirituality into their care. Therefore, it is necessary to identify and record the spiritual needs of people with advanced dementia, as well as to design specific care programs, such as spiritual reminiscence.

Let me end this contribution with another story. This happened just after the first lockdown in June 2020, when I was again allowed access to the dementia ward (unlike many other chaplains who were considered non-essential and were asked to stay away from the residences).

The old man says he is a practising Catholic. “God is important,” he tells me, because “for young people it gives support.” When I ask him if God also supports older people, he pouts. He is much more doubtful about God's supportive presence alongside the elderly!

In the evening, I meet the old man’s daughter and I make her listen to a recording of my conversation with her father. She is surprised by the richness of our exchange. “I don't talk to my father”, she tells me, “I ask him if the meal was good, he answers yes or no, and that's all.” When I ask her how she experienced the lockdown, she sighs: “For me, it was very difficult. For several weeks I had no news from my father, so I had to completely trust the medical team, but I had nothing tangible from him.”

After three months of lockdown, she finds her father has changed a lot. He walks with ever greater difficulty and shows little interest in anything. “I'm afraid to bring him home for lunch on Sunday,” she says. What would she do if it started all over again? Her answer is unequivocal: “I would sign any release to continue to see him. I don't want to shock you, but for my dad, I would wish for a shorter life if necessary, but a life where there are relationships, where we come into contact, where we see each other, where we touch each other. I mean, life doesn't make sense otherwise.”

At our next meeting I ask the old man what gives him hope? He reflects a long while: “What gives me hope is reciprocity in relationships. For the future, I hope to see families reunited.”

And I cannot help but wonder: coming just after a full three months of lockdown, does not his reflection sound like a deeply human, and also a spiritual, desire for love? And is it not good medical ethics to acknowledge and address all the deeply human dimensions of patients?

## Footnotes

1 This contribution is adapted and translated from an article published in *Revue d’éthique et de théologie morale* in 2021 [1].

2 A Mini-Mental State Examination (MMSE) is a set of questions that healthcare professionals use to check for cognitive impairment. The maximum score is 30. A MMSE is not the same as a medical decision-making capacity assessment.

3 It is precisely this type of reasoning that led the Dutch courts to decide that euthanasia on persons with dementia is authorized, when it is asked for in their advance care planning, even if in their present state these persons express not wanting to die. In its verdict of 2019 (par 5.3.2) the court stated: “The court is further of the opinion that, given the fact that the patient could no longer coherently express her will, the defendant did not have to see any contraindications in the patient's contradictory statements regarding her wish to live or, on the contrary, to die.” See [7].

4 Réseau Santé, soins, spiritualités.

5 Translation: “We do not talk about that”.

6 Psychological care differs from spiritual care in that it has a therapeutic goal – it aims to cure a person who is ill. Spiritual care is more like poetry, music, love, and prayer. It needs to be taken care of but does not imply the idea of a “cure”.
